# From bench to bedside: Calprotectin (S100A8/S100A9) as a biomarker in rheumatoid arthritis

**DOI:** 10.3389/fimmu.2022.1001025

**Published:** 2022-11-03

**Authors:** José Inciarte-Mundo, Beatriz Frade-Sosa, Raimon Sanmartí

**Affiliations:** ^1^ Biological aggression and Response Mechanisms, Inflammatory joint diseases (IJDs), Institut d’Investigacions Biomèdiques August Pi i Sunyer (IDIBAPS), Hospital Clinic, University of Barcelona, Barcelona, Spain; ^2^ Rheumatology Department, Hospital Clinic, University of Barcelona, Barcelona, Spain

**Keywords:** calprotectin, rheumatoid arthritis, biomarker, acute phase reactants, CRP - C-reactive protein

## Abstract

S100A9/S100A8 (calprotectin), a member of the S100 protein family, has been shown to play a pivotal role in innate immunity activation. Calprotectin plays a critical role in the pathogenesis of rheumatoid arthritis (RA), as it triggers chemotaxis, phagocyte migration and modulation of neutrophils and macrophages. Higher calprotectin levels have been found in synovial fluid, plasma, and serum from RA patients. Recent studies have demonstrated better correlations between serum or plasma calprotectin and composite inflammatory disease activity indexes than c-reactive protein (CRP) or the erythrocyte sedimentation rate (ESR). Calprotectin serum levels decreased after treatment, independently of the DMARD type or strategy. Calprotectin has shown the strongest correlations with other sensitive techniques to detect inflammation, such as ultrasound. Calprotectin independently predicts radiographic progression. However, its value as a biomarker of treatment response and flare after tapering is unclear. This update reviews the current understanding of calprotectin in RA and discusses possible applications as a biomarker in clinical practice.

## 1 Introduction

Rheumatoid arthritis (RA) is a heterogeneous disease of unknown origin, characterized by chronic polyarthritis that may lead to joint destruction, disability, and increased mortality. Extraarticular manifestations are not uncommon. RA affects 0.5-1% of the adult population, predominantly females. Genetic and environmental factors have been implicated in the susceptibility to RA. Autoimmunity, with the presence of characteristic autoantibodies such as rheumatoid factor or anticitrullinated peptide autoantibodies, are implicated in the pathogenesis of RA, although recent data confirms the role of the innate immune system in this disease (1).

The innate immune system plays a central role in initiating local inflammation, contributing to the pathogenesis of RA by promoting the production of inflammatory cytokines and chemokines. Pattern recognition receptors (PRRs) are a family of receptors of the innate immune system that bind to damage-associated molecular pattern molecules (DAMP) (2). The most important PRRs are Toll-like receptors (TLRs), which allow the activation of monocytes, neutrophils, dendritic cells, natural killer (NK) cells and B cells (3).

S100A9/S100A8 (calprotectin) a member of the S100 protein family has been studied as an important proinflammatory factor of innate immunity as an endogenous DAMP *via* TLR4 activation. Calprotectin plays a critical role in the development of inflammation loops in RA as a trigger for chemotaxis, phagocyte migration and modulation of various macrophage functions (4–6).

This update reviews the current understanding of calprotectin in RA and discusses possible applications as a biomarker in clinical practice.

## 2 Calprotectin and the S100 protein family

The first members of the S100 protein family were purified from bovine brain in the early 1980s. The protein complex was denominated “S100” because of its 100% solubility in ammonium sulphate solution (7). The S100 protein family is specifically linked to innate immune functions by their expression in cells of myeloid origin.

The S100 protein family is widely expressed, although they are not ubiquitous, and several have highly restricted distributions. The functions of these proteins vary widely between individual members, functioning as both intracellular and extracellular signaling molecules. S100 protein functions include cytoskeletal function, homeostasis, tumor-suppression, antimicrobial response, chemotactic activity, atherogenesis and protection from oxidative cell damage in brain tissue. The main tissue cell locations and functions are summarized in **Table 1**.

**Table 1:** S100 protein family. Main tissue cell localization and function of S100 protein family.

S100 protein	Tissue and subcellular location	Function
**S100A1**	It is expressed in skeletal muscle fibers, cardiomyocytes and some neuronal populations (8)	Accelerates deterioration of cardiac performance and transition to heart failure (9)
**S100A2**	It is expressed in the lung, kidney, prostate, skin and salivary and mammary glands (10)	Tumor-suppressing function (11)
**S100A3**	Highly expressed in hair root cells and some astrocytomas (12).	Tumor-suppressing function and epithelial cell differentiation (11)
**S100A4**	It is overexpressed in breast cancer, gastric cancer and non-small cell lung cancer (NSCLC) (13)	Apoptosis, cell motility, and tumorigenesis (14)
**S100A5**	It is upregulated in bladder cancers and recurrent grade I meningiomas (15)	Not described
**S100A6**	It is overexpressed in lung, bile duct, and brain cancer, and non-small cell lung adenocarcinoma (16–19)	Cell proliferation, cytoskeletal dynamics and tumorigenesis (20, 21)
**S100A7**	It is overexpressed in inflammatory skin diseases. lymphocytes, monocytes and granulocytes (22)	Antimicrobial response, chemotactic activity, prevent generation of amyloidogenic peptides in Alzheimer’s disease. psoriasis, and atopic dermatitis (23–25).
**S100A8**	It is found in macrophages, dendritic cells, microvascular endothelial cells but not endothelial cells from larger vessels, epithelial cells (e.g., keratinocytes) and fibroblasts (26)	Regulation of inflammation. Effect on leukocyte adhesion and neutrophil adhesion (e.g., asthma) (5).
**S100A9**	It is found in myeloid cells: (macrophages and neutrophils) some cancer cells (e.g., breast cancer, esophageal squamous cell carcinoma) (27).	Leukocyte migration, adhesion and transmigration from blood vessels, thus it has anti-inflammatory properties. In some cancer cells it promotes growth suppression (5).
**S100A10**	It is found in cell types throughout the body though it is located predominantly in the lungs and kidneys (28).	It is involved in the trafficking of proteins to the plasma membrane (angiogenesis and endothelial cell function) (28) and can be expressed on the cell surface as a receptor with clinical implications in some malignancies (e.g., tumor migration, hemorrhagic phenotype of promyelocytic leukemia) (29).
**S100A11**	It is induced/released by chondrocytes (30)	Stimulates cell growth by enhancing the level of epidermal growth factor (EGF) family proteins, promoting hypertrophic chondrocyte differentiation (31).
**S100A12**	It is constitutively expressed in neutrophils and inducible in macrophages and smooth muscle cells. it is expressed in human aortic aneurysms (32).	Expression in epithelial cells is associated with growth arrest. Potentiation of atherogenesis (33).
**S100A13**	It is found in multiple cell types, including fibroblasts, osteoblasts and melanoma cells (20)	It may play a pivotal role in angiogenesis (34).
**S100A14**	It can be found in an esophageal squamous cell carcinoma cells (35).	It may function as a cancer suppressor, playing a dual role in tumor cells (enhancing or decreasing tumor cell invasiveness) (36).
**S100A15**	It is expressed in keratinocytes in inflamed skin (37).	Antimicrobial activity against E. coli (38).
**S100A16**	It is expressed in theca cells, urothelial cells, pancreatic endocrine cells, prostatic glandular cells, squamous epithelial cells, basal prostatic cells, and suprabasal keratinocytes. It is upregulated in several tumors (39).	Recently, a role in insulin sensitivity regulation has been described (40).
**S100B**	It is expressed in astrocytes, certain neuronal populations, Schwann cells, melanocytes, chondrocytes, adipocytes, skeletal myofibers and associated satellite cells, certain dendritic cell and lymphocyte populations and other cell types (41).	It induces neurogenesis and reduces delayed neuronal injury and might contribute significantly to neuroinflammation (42).
**S100P**	It can be expressed in urothelial cells, pancreas, syncytiotrophoblasts, gastric mucus-secreting cells (43)	It promotes transendothelial migration of tumor cells and, potentially, metastasis (44).
**S100Z**	The highest levels were found in the spleen and leukocytes, and in some tumor tissues (e.g., prostate) (45).	Tumor growth. Recently, it has been associated with pulmonary systemic sclerosis (45).

Among the S100 protein family, calprotectin (S100A8/S100A9) is primarily expressed in innate immune cells, particularly in neutrophils, monocytes, and macrophages, which constitute approximately 40% of the cytosolic proteins in these cells. However, under specific stimuli, calprotectin may be expressed in other cell lines, such as osteoclasts and keratinocytes (46–48). The gene encoding calprotectin subunits is located in the gene cluster on human chromosome 1q12-1q21 (49, 50).

Although calprotectin has been recognized for almost 40 years, it has only recently become of interest as a biomarker of disease activity and damage in rheumatology and was previously known as L1 protein (51), cystic fibrosis-associated antigen (CFA) (52), calgranulins A and B (53), S-1OOa and b (54) or myeloid related protein 8 and 14 (MRP 8/14) (48). Calprotectin is a well-established biomarker in other medical areas, such as calprotectin in faeces in inflammatory bowel disease (55–59).

### 2.1 Calprotectin molecular structure

Calprotectin is a calcium- and zinc-binding heterodimeric molecule of 36.5 kDa, with two heavy and one light chain non-covalently linked (51). It has two subunits, S100A8 and S100A9, which are 8.3 kDa and 13.3 kDa, respectively (60). S100A8 is the active subunit and S100A9 acts as the regulatory subunit, preventing early degradation of S100A8 (61). The EF-hand is composed of two α-helices flanking a central calcium-binding loop, resulting in classical helix-loop-helix domains (62). Calprotectin is mostly found in the form of heterodimers (S100A9-S100A8) and tetramers (S100A9-S100A8)2 in a calcium dependent-manner (63).

Two independent calprotectin activation pathways have been proposed to explain calprotectin release to extracellular compartments. The first, a canonical pathway, includes protein kinase C, which is induced by different inflammatory stimuli (e.g., bacteria). The second, a non-classical secretion avoiding the Golgi-associated pathway, requires the elevation of intracellular calcium levels, induced by contact between phagocytes and pre-activated endothelial cells by tumor necrosis factor (TNF), resulting in active secretion of calprotectin by phagocytes (63). Recently, a new activation mechanism has been proposed, namely activation *via* chromatin in neutrophil extracellular traps (NETs) (64). In addition, calprotectin may be secreted passively from apoptotic cells (61).

### 2.2 Calprotectin functions

#### 2.2.1 Intracellular functions

**Figure 1:**
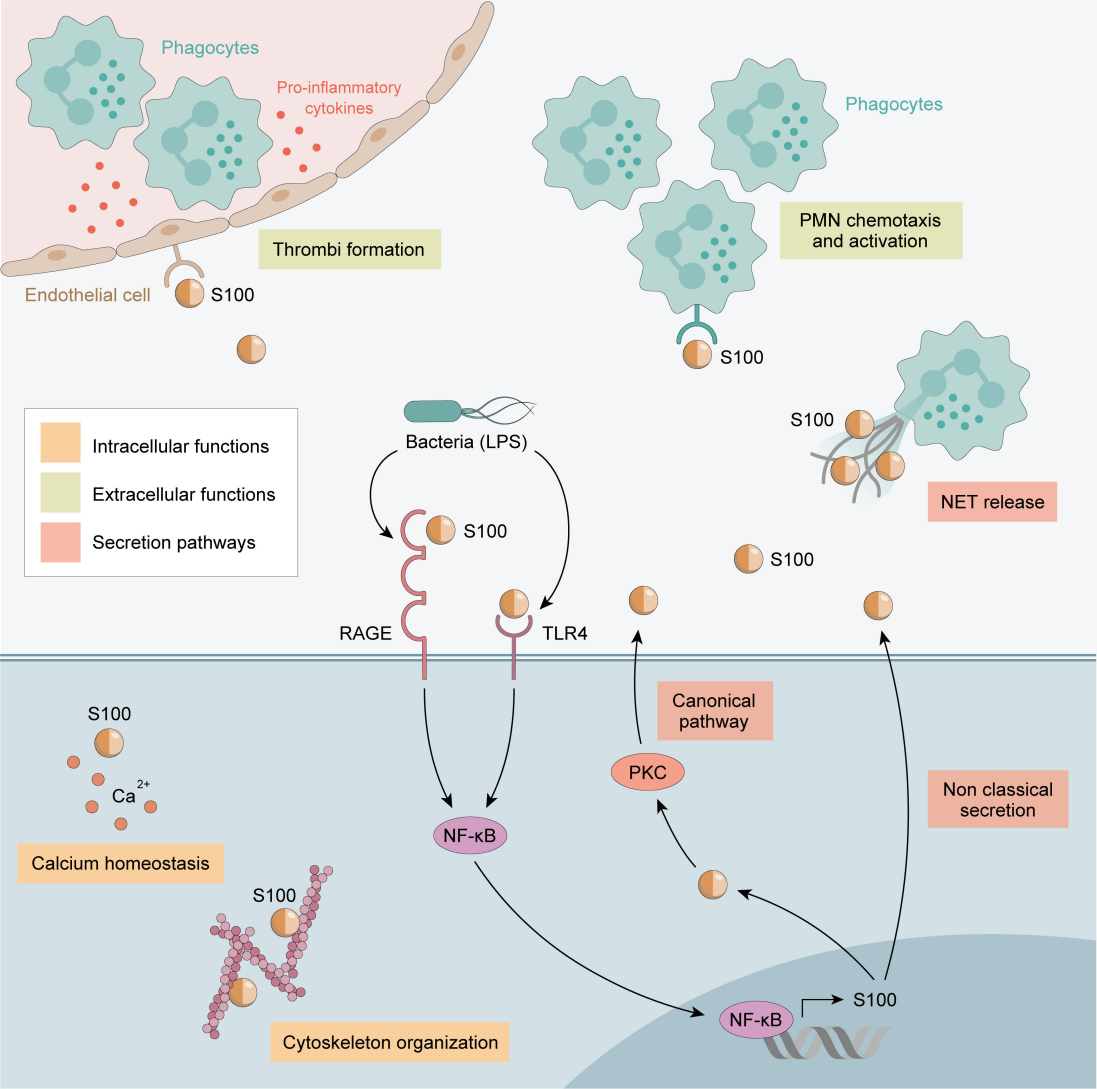
Calprotectin Functions. Intracellular functions are shown in orange and extracellular functions in green. Calprotectin Intracellular functions includes cytoskeleton cell migration and calcium homeostasis. Extracellular functions involve endothelial cells activation, promoting the adhesion of phagocytes to the vascular endothelium and thrombi formation. Also increase chemotaxis and the activation of PMN. Finally, calprotectin exerts a strong antimicrobial action against a variety of bacterial and fungal pathogens.

Calprotectin complexes are known to interact with cytoskeletal components such as actin filaments, keratin, vimentin, and microtubules in a calcium-dependent manner. High calcium concentrations induce a rearrangement of calprotectin into tetramers, allowing translocation to the cell membrane and tubulin polymerization (61). This process is regulated by the phosphorylation of the threonine at position 113 in S100A9 by p38 MAPK (**Figure 1**).

#### 2.2.2 Extracellular functions

Extracellular calprotectin complexes interact with endothelial cells by binding to heparan sulfate and, specifically, carboxylated glycans, and up-regulate integrin receptors on leukocytes, resulting in the activation of endothelial cells. Activated endothelial cells express a pro-inflammatory cytokine profile (e.g., IL-1, IL-8 MCP-1), and thus calprotectin plays a central role in promoting the adhesion of phagocytes to the vascular endothelium and thrombi formation (65, 66). Calprotectin generates a positive feedback loop, increasing chemotaxis and the activation of PMN, which are the main source of calprotectin: therefore, calprotectin has an autocrine and paracrine function (67). Notably, calprotectin interaction with non-activated endothelium inhibits its secretion, meaning calprotectin is only released at sites of inflammation by activated phagocytes (**Figure 1**).

Calprotectin exerts a strong antimicrobial action against a variety of bacterial and fungal pathogens. Calprotectin recognizes proteins related to bacteria, such as lipopolysaccharides (LPS), up-regulating the production of pro-inflammatory profile cytokines, such as TNF-α, IL-1β, and IL-12 locally (68, 69). It is recognized as an endogenous DAMP and binds to the TLR4 receptor and RAGE, amplifying the innate immune response and inducing PMN recruitment to inflamed tissues (3). The antibacterial activity of calprotectin results from the sequestering of transition metals by chelation of nutrient Mn^2+^ and Zn^2+^ (70). Engulfment of bacteria by macrophages leads to decreased Zn^2+^ uptake and increased Zn^2+^ efflux from the cytoplasm and the efflux of Mn^2+^ and Fe^2+^ from the phagosome by NRAMP family transporters (71). Chelation is mediated through two high-affinity binding sites, both of which can bind Zn^2+^ with nanomolar affinity, while only one binds Mn^2+^ with this affinity (71).

Calprotectin also activates the MyD88-dependent and TIR domain-containing adaptor protein inducing IFNβ (TRIF; also known as TICAM1)-dependent signaling pathways downstream of TLR4, resulting in NF-κB-mediated and interferon regulatory factor (IRF)-mediated gene transcription. EMMPRIN, a transmembrane glycoprotein of the immunoglobulin superfamily is also able to bind S100A9. However, its biological function has not yet been described (72).

### 2.3 Calprotectin in health and disease

Studies have shown that calprotectin levels are minimal in serum and stool samples from healthy population compared with patients with inflammatory conditions (73–76). High calprotectin serum levels were also observed in patients with infectious disease and sepsis (77). Recently, the accuracy of calprotectin as a biomarker of bacterial respiratory disease was found to be even higher than procalcitonin (78).

Increased calprotectin expression has been found in patients with rheumatic diseases (See **Table 2**). Higher calprotectin levels have been found in plasma, serum and faecal samples from patients with RA (100), spondyloarthropathies (SpA) (101), inflammatory bowel disease (IBD) (102) and type 2 diabetes (T2D) (103). In IBD, faecal calprotectin has been shown to be a more sensitive indicator of inflammatory activity and is being used for both the diagnosis and follow-up in routine clinical practice (104). Calprotectin levels are not affected by age or gender (105).

**Table 2:** Calprotectin in Rheumatic Diseases. This table summarizes the main findings of calprotectin levels and rheumatic diseases other than RA.

Disease	Calprotectin source	Main finding
**Reactive arthritis**	**Plasma and synovial fluid.**	• CLP correlated with CRP and disease activity (79). • CLP was the first biomarker to return to baseline levels after disease improvement (79).
**Ankylosing spondylitis**	**Serum**	• CLP correlated with PGA, pain VAS, BASDAI, BASFI, and ASDAS (80, 81). • CLP is an independent marker for radiographic progression (82).
**Psoriatic arthritis**	**Serum**	• CLP levels are significantly higher in active PsA patients than in healthy controls (83). • Higher CLP levels were found in the polyarticular group than in patients with mono/oligoarticular disease (84). • CLP levels are sensitive to change, and its levels decrease after TNFi and IL17i treatment (83).
**Systemic juvenile idiopathic arthritis**	**Serum**	• CLP levels are sensitive to change (85). • CLP more accurately predicts disease relapse (86).
**Adult onset Still disease**	**Serum**	• CLP levels are significantly higher than RA, SLE patients or controls (87, 88).
**Gout**	**Serum, synovial biopsy and tophi**	• CLP levels were elevated in the synovium, tophi, and serum of patients with gout (89). • CLP levels correlated with disease activity (89).
**Systemic lupus erythematosus**	**Urine**	• CLP levels in SLE-LN patients (90). • CLP levels correlated with disease activity (91).
**Primary Sjogren syndrome**	**Serum, saliva and salivary gland biopsy.**	• Increased levels in pSS patients (92, 93).
**Systemic sclerosis**	**Serum, broncho-alveolar lavage fluid, skin biopsy**	• Increased levels in SSc patients (94, 95). • Increased levels in diffuse cutaneous SSc patients (96).
**Behcet’s disease**	**Serum**	• CLP levels are significantly higher than healthy controls (97). • CLP correlated with CRP and disease activity (97)
**Idiopathic inflammatory myopathies**	**Serum and muscle biopsy**	• Increased levels in IIM patients (98). • CLP promotes myoblast activation (99).

CLP, calprotectin; PGA, patient global assessment; pain VAS, pain visual analogue scale; BASDAI, Bath AS disease activity index; BASFI, the Bath AS functional index; ASDAS, Ankylosing Spondylitis Disease Activity Score; TNFi, TNFn inhibitors; IL17i; interleukin 17 inhibitor; SLE-LN, SLE-lupus nephritis.

Recently, a new disorder, characterized by recurrent infections, hepatosplenomegaly, anemia, cutaneous vasculitis, and evidence of systemic inflammation has been described. These patients have shown hyperzincemia with hypercalprotectinaemia (106).

## 3 Calprotectin in rheumatoid arthritis

There is abundant evidence that the innate immune system is persistently activated in RA, as predominately macrophages are found in rheumatoid synovium (107).

**Figure 2:**
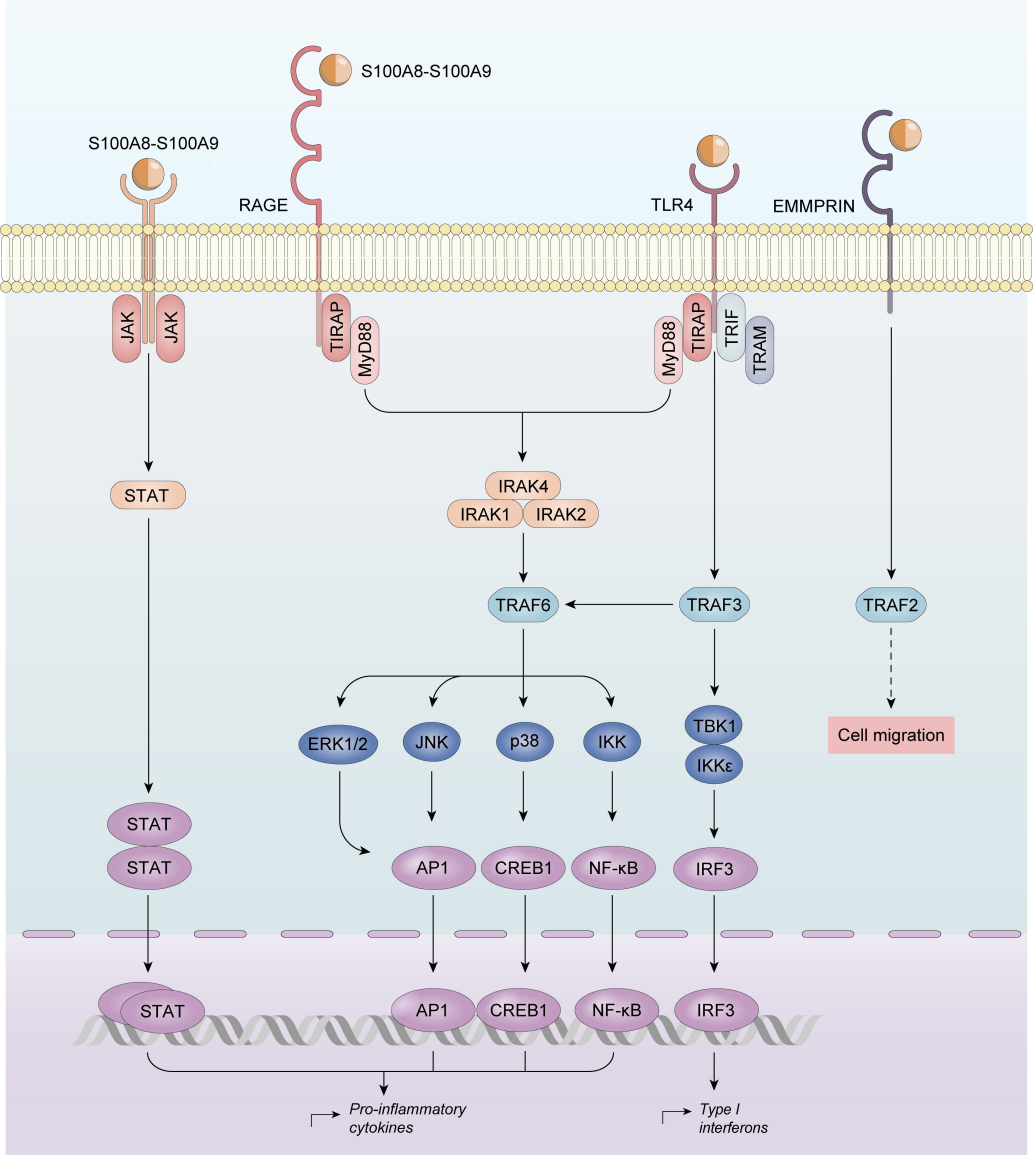
Calprotectin activation in Rheumatoid Arthritis. Calprotectin activates nuclear factor κB (NF-κB), and protein kinase-mediated signal transduction pathways, including c-Jun-N-amino-terminal kinase (JNK), extracellular-regulated kinase 1/2 (ERK1/2), and Janus kinase/signal transducers and activators of transcription (JAK/STAT) upregulating proinflammatory cytokines production.

In RA, calprotectin induces nitric oxide synthase (iNOS) by nuclear factor *κ*B (NF-*κ*B) activation (108). Calprotectin allows the phosphorylation of multiple protein kinase-mediated signal transduction pathways, including c-Jun-N-amino-terminal kinase (JNK), extracellular-regulated kinase 1/2 (ERK1/2), and Janus kinase/signal transducers and activators of transcription (JAK/STAT) (109). However, calprotectin activation converges in multiple pathways whose activation enhances the production of proinflammatory cytokines, namely tumor necrosis factor-*α* (TNF-*α*), IL-6, IL-8, IL-12/23, and IL-18 (110), which are known to be physiopathologically- and clinically-relevant in RA (**Figure 2**).

Calprotectin expression is noted predominantly in the macrophages of the synovial lining layer in tissues adjacent to the cartilage-pannus junction (CPJ), suggesting altered activation and differentiation of lining layer macrophages at the CPJ, which is the site of maximum cartilage destruction in RA (111). Calprotectin is synthesized in fibroblast-like synoviocytes (FLSs), which are crucial players in the pathogenesis of synovitis in RA. IL-22 enhanced FLS proliferation and up-regulated MMP1 and S100A8/A9 production (112). Interestingly, when stimulated FLSs were treated with a JAK 2 and JAK3 inhibitor, there was a significant decrease in IL-22-induced S100A8/A9 production.

Calprotectin is released from activated leukocytes leading to increased concentrations in RA plasma and serum (113, 114). a study focused on determining the association of calprotectin in EDTA-plasma or in serum with disease activity, found stronger plasma associations with all measures of disease activity (115). However, ELISA commercial kits are designed to determine both, and most evidence available is drive by from serum determinations.

### 3.1 Calprotectin as a biomarker of rheumatoid arthritis

Higher calprotectin levels have been found in synovial fluid (SF), plasma and serum from RA patients (113, 114). A recent study demonstrated that the most enhanced proteins in RA SF were the S100A8, S100A9 and S00A12 proteins, using proteomic fingerprints of RA patients’ serum (116). A German study found that calprotectin and other S100 proteins were the most up-regulated proteins in SF in RA patients. Their expression was about 10-fold higher than that observed in the SF of osteoarthritis patients (OA). Not only was calprotectin the best RA biomarker identified in this study, but higher calprotectin levels were found in the SF of RA compared with SpA patients: therefore, calprotectin may differentiate RA from other inflammatory arthritis (117).

Interestingly, calprotectin levels correlated with rheumatoid factor. Furthermore, higher calprotectin plasma levels were found in seropositive than in seronegative patients. The correlation with ACPA titres remains unclear (118–126).

Calprotectin also significantly contributes to comorbid conditions in RA patients, such as cardiovascular disease. High calprotectin levels have been associated with precocious atheroma formation and the accelerated development of atherosclerosis (127).

In summary, serum calprotectin levels may provide information on macrophage activation, supporting their potential role as a biomarker of disease activity, radiographic progression and therapeutic response.

### 3.2 Calprotectin as a marker of disease activity

In RA, changes in the amount of synovial sublining macrophages correlate with disease activity (128): these macrophages are the major source of calprotectin, and thus their levels may provide reliable information on their activation 74). Recent studies have demonstrated better correlations between calprotectin and DAS28, swollen joint count (SJC), CDAI, SDAI and physical global VAS than C-reactive protein (CRP) or the erythrocyte sedimentation rate (ESR) (121–123, 129). This was especially pronounced in patients receiving IL-6 inhibitors, with a pronounced decrease in CRP serum levels independently of disease activity (129). These correlations were independent of the disease stage. Studies in both recent-onset patients (105, 130) and those with established disease (123) have demonstrated a significant correlation between calprotectin and SJC, DAS28, SDAI and CDAI (73, 75, 76, 131, 132). In a recent metanalysis of 16 studies, the relationship between calprotectin and disease activity was confirmed (74).

In clinical remission, CRP and ESR may be normal, although there may be residual inflammatory activity, especially in patients receiving biological therapy. Calprotectin levels, but not CRP or ESR levels, were significantly lower in patients with no swollen joints than in those with ≥ 1 swollen joint, supporting the hypothesis that calprotectin levels reflect local ongoing inflammation rather than a systemic inflammatory response (118, 120, 126). Studies by our research group have demonstrated that calprotectin more accurately stratifies disease activity in RA patients in remission or low disease activity receiving TNF inhibitors (133) or the biologic agent, tocilizumab (129). This effect might lead overestimates of the response rate when disease activity indices including ESR or CRP are used (134). In these patients, calprotectin, but not CRP or ESR, can distinguish between patients without swelling from those with ≥1 swollen joint. Calprotectin serum levels, but not CRP, are independent of trough serum tocilizumab levels (129). Recently, these results have been replicated in an independent large cohort (135).

RA patients with normal CRP pose a therapeutic challenge in daily clinical practice. Therefore, calprotectin may have a potential role in assessing disease activity in patients with remission or low disease activity, identifying patients with subclinical synovitis more accurately.

#### 3.2.1 Calprotectin serum levels are sensitive to change

Calprotectin serum levels decreased after treatment, independently of the DMARD type or strategy (105). A significant reduction in serum calprotectin levels was observed after three months of csDMARD treatment (105, 123, 136). The same occurs during biological therapy. Infliximab significantly decreases serum calprotectin levels in RA patients, as confirmed by immunohistochemical staining for S100A8 on serial synovium sections, which showed a progressive decrease in the number of infiltrating S100 A9-positive macrophages. Similar results were observed in RA patients receiving adalimumab and etanercept (105, 137–139).

#### 3.2.2 Calprotectin and ultrasound synovitis

Musculoskeletal ultrasound (US) is a non-invasive diagnostic technique widely used in rheumatology to assess joint inflammation with greater sensitivity (140). A pilot study explored the associations between calprotectin and comprehensive US examination in 20 RA patients starting treatment with adalimumab and found a significant association with B-mode and the power Doppler score, and there was a correlation between calprotectin and the number of swollen joints (141) and the results were recently replicated (132, 142). In a one-year prospective cohort study of patients with established RA patients, calprotectin showed the overall strongest correlations with US scores and SJC, even after adjustment for several variables (143). We found that RA and PsA patients in remission or low disease activity with a US power Doppler signal had significantly-higher calprotectin levels than those without, and calprotectin correlated better with US power Doppler, synovial hypertrophy and US global scores than ESR or CRP (144). Taken together, serum calprotectin and power Doppler are both identifying local active synovitis in patients with inflammatory arthritis, even those with low levels of disease activity.

#### 3.2.3 Calprotectin and radiographic progression.

Murine arthritis models have shown that overexpression of IL-17 and TNFα strongly enhanced up-regulation of calprotectin, resulting in bone erosion. In contrast, calprotectin deficiency in mice protected against the IL-17/TNFα effect in cartilage (145).

In RA, calprotectin antigens were located in the synovial cartilage, suggesting a pivotal role in cartilage destruction and subchondral bone erosions, a typical hallmark in active RA patients (111). In this regard, a study of 145 RA patients showed that baseline calprotectin levels were independently associated with the van der Heijde modified Sharp score (SvH) and the Rheumatoid Arthritis Articular Damage Score (RAAD score), even when adjusted for CRP, ESR, rheumatoid factor, DAS28, sex and age (123). The prospective follow up of this cohort found that calprotectin was an independent predictor of radiographic joint damage after 10 years. Patients with normal baseline calprotectin levels had less joint damage; again, calprotectin was independently associated with progression in the SvH and RAAD scores (125). Similarly, a longitudinal study with a median of 8-years of follow-up, demonstrated that calprotectin predicts erosive disease and joint space narrowing (73). An exploratory analysis has correlated calprotectin significantly with joint bone marrow edema on MRI in RA patients in clinical remission (146). Recently, the same results have been replicated in a large early-RA cohort from the ARTIC trial, where high levels of calprotectin were associated with radiographic progression in multivariate models (130).

### 3.3 Calprotectin and treatment response

Prediction of the individual response to treatment has become a major clinical challenge in RA. Recent studies and *post-hoc* clinical trials have provided evidence of calprotectin’s accuracy in predicting the response to csDMARD and bDMARD therapy in RA. In patients receiving csDMARDs, the results are unclear, and decreases in serum calprotectin levels, rather than CRP, were associated with improvements in the SJC over time (105). Patients who achieved remission had a significant reduction in serum calprotectin levels. Likewise, a study shown that baseline serum calprotectin levels decreased rapidly in responders after csDMARD treatment but remained stable in non-responders (122). In early RA, a *post-hoc* analysis of the prospective ESPOIR cohort found that calprotectin was not an independent predictive factor of the response to MTX. Although the study excluded a large proportion of cohort participants, its results suggested a potential interest in calprotectin as a part of a multivariable score for personalized medicine in these patients (147). Similar results have been seen in RA patients receiving biological therapy: a prospective cohort study evaluated 170 RA patients receiving biological therapy (adalimumab, infliximab, and rituximab). Calprotectin levels were measured at 0, 4 and 16 weeks after biologic drug initiation. As previously described, responders had higher baseline calprotectin levels than non-responders. Higher baseline calprotectin levels increased the odds of being classified as a responder by up to 55-fold, and levels decreased after treatment. In contrast, non-responders had stable calprotectin levels during the study (148). The authors developed a treatment algorithm based on a prediction score using calprotectin and concluded that it may have potential in personalized treatment in RA (149).

Calprotectin levels at baseline were associated with biological treatment survival (150). A prospective cohort study did not find that baseline calprotectin levels were predictive of the response after 6 months of treatment, although a significant decrease in serum levels was observed in responders (122). A recent study demonstrated a significant decrease in calprotectin in the first month of biological therapy, which was predictive of the EULAR response at 3,6 and 12 months (143).

A recent systematic review summarized the results from 17 studies including 1065 patients and found that calprotectin levels decreased after treatment, although there was a wide range of levels and marked interstudy and intrastudy variability. Baseline calprotectin levels were a significant and independent predictor of erosive progression and therapeutic responses, particularly in patients receiving biological treatment (151).

Taken together, this data supports the idea that calprotectin could potentially help to monitor disease activity and predict the response to bDMARDs in RA, although there is no data available on patients receiving DMARDs. Randomized trials are needed to define the role of calprotectin as a predictor of treatment response, but there is a potential role for calprotectin in the follow-up of RA patients.

### 3.4 Calprotectin and disease relapse

A clinically relevant unmet need is stratification of the risk of relapse in RA patients in remission or low disease activity. Data demonstrate that calprotectin levels are increased during relapse (152). A prospective one-year follow up cohort study found that calprotectin levels strongly and independently predicted disease relapse in RA with low levels of disease activity during TNFi treatment. In contrast, a prospective cohort study analyzing the role of calprotectin in predicting flares in RA with low disease activity by DAS28 (DAS28<3.2) found that only HAQ-DI remained a significant independent predictor of flares in the multivariate analyses. At the time of the flare, DAS28 and its components significantly correlated with calprotectin, but the correlation was low, suggesting a non-inflammatory component in most events (153).

Another situation is the risk of flare in RA patients before tsDMARD tapering. The capacity of calprotectin in predicting flares in patients undergoing tapering of biologics has been assessed. Calprotectin levels were determined in serum samples from participants in two prospective studies (DRESS and BIO-TOP). Although calprotectin has some predictive value for the clinical response after starting anti-TNF treatment, it has no added value for other clinical factors (154). In contrast, analyses from two tapering studies (IMPROVED study and the RETRO study) showed that calprotectin levels in remission on DMARDs are higher in patients who will flare upon DMARD tapering/cessation (155).

The definitive role of calprotectin as a predictor of disease relapse remains unclear, as there are no specific randomized clinical trials to assess its potential use.

### 3.5 Calprotectin inhibition as therapeutic approach in RA

Based on the fundamental role of calprotectin in the modulation of acute and chronic inflammation, its inhibition could be a novel target in the treatment of RA. An experimental study investigated the effects of calprotectin inhibition in RA using neutralizing monoclonal antibodies in a mouse collagen-induced arthritis (CIA) model, Murine S100A9 monoclonal antibody and anti-TNFα treatment were compared. Mice treated with anti-S100A9 showed markedly decreased arthritis severity scores compared to the isotype control group. Overall, anti-S100A9 treatment led to an approximately 50% reduction in disease activity, and preserved bone/collagen integrity. No significant differences in disease activity were observed between anti-S100A9 and anti-TNFα-treated animals, suggesting calprotectin might be a novel therapeutic target in RA (156, 157).

## 4 Discussion and conclusions

There is still an unmet need for robust biomarkers to objectively monitor inflammatory activity and response to therapy in RA and other immune mediated diseases. Based on the pivotal role of calprotectin in the pathophysiology of acute and chronic inflammation, calprotectin blood levels could be a potential biomarker of disease activity in inflammatory arthritis. In RA, there is growing evidence to support the idea that calprotectin more accurately stratifies disease activity than CRP and ESR. Furthermore, recent data has shown that calprotectin serum levels are a potential tool for monitoring disease activity and the therapeutic response in patients receiving biological therapy. However, larger studies and assay standardization are needed in RA patients to ascertain the role of serum calprotectin as a useful biomarker for monitoring disease activity or response to therapy in clinical practice, as occurs with fecal calprotectin in inflammatory bowel disease. Future applications may include potential therapeutic targets, prediction of the response to treatment, or dose-titration of biologics in a personalized medicine approach.

In conclusion, calprotectin plays a pivotal role in innate immune system activation, increasing chemotaxis and the activation of PMN, promoting the production of inflammatory cytokines and chemokines, and contributing to RA pathogenesis. There is growing evidence to support its higher accuracy in stratifying disease activity than CRP and ESR. Calprotectin has shown the strongest correlations with other sensitive techniques to detect inflammation, such as ultrasound. However, its value as biomarker of treatment response and flare after tapering still need larger, standardized studies.

## Author contributions

JI-M: investigation, resources, writing (original draft preparation, review and editing). BF-S: investigation, resources, writing (original draft preparation, review and editing). RS: investigation, resources, writing (original draft preparation, review and editing), supervision, and project administration. All authors contributed to the article and approved the submitted version.

## Acknowledgments

The authors acknowledge David Buss for technical advice, and Antonio García, PhD for Scientific Illustration.

## Conflict of interest

JI-M has received honoraria from AbbVie employee.

The remaining authors declare that the research was conducted in the absence of any commercial or financial relationships that could be construed as a potential conflict of interest.

## Publisher’s note

All claims expressed in this article are solely those of the authors and do not necessarily represent those of their affiliated organizations, or those of the publisher, the editors and the reviewers. Any product that may be evaluated in this article, or claim that may be made by its manufacturer, is not guaranteed or endorsed by the publisher.
